# Editorial

**Published:** 1846-02

**Authors:** 


### THE MEDICAL EXAMINER.

PHILADELPHIA, FEB., 1846.

### EPIDEMIC SMALL-POX.

We continue to hear of the prevalence of this disease in vaiious parts of the country, and now that it is fairly enthroned, we suppose it will reign while subjects last and cold weather continues. In Philadelphia it is evidently on the decline. The number of deaths by it, reported by the Board of Health last week, was but eighteen, which is fifty per cent less than a few weeks back. In other respects our city is as healthy as usual at this season of the year; and notwithstanding the exaggerated reports which have gone abroad, the number of deaths from small-pox, considering how many persons have laboured under it who were not protected by vaccination, has really been very small out of so large a population.

### NEW MEDICAL SCHOOLS.

A *Medical University* (!) for teaching how to steam and give *Lobelia* and *Number Six*, has recently been chartered by the State of Alabama, and a new Medical School at Memphis, by the State of Tennessee. We also learn there are applications pending before the Legislature of Pennsylvania for the establishment of two new ones in Philadelphia, and that one or two more are getting ready to apply! How many more are in embryo, here and elsewhere, we know not. If the people were as wise as Æsop's frogs, they would probably say, "this may be sport to you, gentlemen Professors, but it is death to us."

### THE WEATHER.

The weather in Philadelphia the present winter, as it regards temperature, has thus far been pretty much like the Irishman's pig—"a streak of fat and a streak of lean." We have had three or four spells almost cold enough for a Siberian winter, with intervals that would not discredit the West Indies. At the present moment (January 26, 10 o'clock, P. M.) the mercury in Fahrenheit is at 40° in the open air, with a north eastern exposure, while the streets are covered with smoking ice, which descended a few days since, in the form of hail, rain and snow!

*Introductory Lectures.*

This is the season for the publication of the Introductory Lectures delivered at the Medical Schools throughout the country. Among the offerings of this kind, we notice the following on our table:

"An Introductory Lecture delivered before the Class of Institutes of Medicine, in Jefferson Medical College, November 3, 1845. By ROBLEY DUNGLISON, M. D. Published by the Class."

"Introductory Lecture to the course of General, Descriptive and Surgical Anatomy, in Jefferson Medical College. Delivered November 5, 1845. By JOSEPH PANCOAST, M. D. Published by the Class."

"Sources, Evils, and Correctives of Professional Discontent. An Introductory Lecture, delivered November 4, 1845. By JOHN P. HARRISON, M. D. Professor of Materia Medica and Therapeutics in the Medical College of Ohio."

"The Reciprocal Obligations of Professors and Pupils. An Introductory Lecture, delivered by THOMAS D. MITCHELL, M. D. Professor of Materia Medica and Therapeutics, and Lecturer on Obstetrics, in Transylvania University, November 3, 1845. Published by the Class."

"An Introductory Lecture, delivered by GUNNING S. BEDFORD, A. M., M. D., Professor of Midwifery and the Diseases of Women and Children, in the New York University. Session 1845–6. Published by the Class."

---

*Catalogues and Annual Announcement.*

We have received the following Catalogues and Annual Announcement.

"Catalogue of the Officers and Students of Yale College, 1845–6." The number of Medical Students, the present Session, is 53.

"Catalogue of the Medical Institution of Geneva College, Session 1845–6." Number of the Class, 178.

"Annual Announcement of the Philadelphia Medical Association, for the Session of 1846." This is a private Association of highly competent gentlemen for giving instruction in the several branches of Medical Science during the summer months. The lectures commence early in April, and continue until the end of October, with the usual recess during summer.

